# Radiation exposure during endoscopic retrograde cholangiopancreatography according to clinical determinants

**DOI:** 10.1097/MD.0000000000019498

**Published:** 2020-03-27

**Authors:** Chi Hyuk Oh, Seok Ho Dong, Jung-Wook Kim, Gi-Ae Kim, Jae Min Lee

**Affiliations:** a Division of Gastroenterology and Hepatology, Department of Internal Medicine, Kyung Hee University College of Medicine; b Division of Gastroenterology and Hepatology, Department of Internal Medicine, Korea University College of Medicine, Seoul, Republic of Korea.

**Keywords:** endoscopic retrograde cholangiopancreatography, fluoroscopy time, radiation exposure

## Abstract

This study aimed to analyze the dose of radiation to which the physician is exposed during endoscopic retrograde cholangiopancreatography (ERCP) and to identify predictive factors of radiation exposure during the procedure. Furthermore, we evaluated the patient characteristics and procedural factors associated with prolonged fluoroscopy time (FT).

A cross-sectional retrospective analysis of 780 ERCPs performed at a tertiary academic hospital over a 2-year period was conducted. The primary outcome was radiation exposure during ERCP as determined by FT; additionally, the association between variables and radiation exposure was determined. Moreover, we evaluated their correlations with age, sex, body mass index (BMI), diagnosis, duration of procedure, procedure name, and procedure complexity.

According to the analysis of the 780 ERCPs performed in 2 years, the mean FT was 5.07 minutes (95% confidence interval [CI], 4.87–5.26). The mean radiation durations were as follows: cholelithiasis, 5.76 minutes (95% CI, 4.75–6.80); malignant biliary obstruction, 6.13 minutes (95% CI, 5.91–6.35); pancreatic disease, 5.28 minutes (95% CI, 4.45–6.28); and benign biliary stricture, 5.32 minutes (95% CI, 5.02–5.94). Significant differences affecting fluoroscopy duration between the 2 endoscopists were not observed in the present study. Multivariate analysis revealed that prolonged fluoroscopy duration was related to specific characteristics, including higher BMI (BMI >27.5 kg/m^2^) (+4.1 minutes; 95% CI, 2.56–5.63), mechanical lithotripsy (+4.85 minutes; 95% CI, 0.45–9.25), needle-knife use (+4.5 minutes; 95% CI, 2.15–6.86), and malignant biliary obstruction (+2.34 minutes; 95% CI, 0.15–4.53).

ERCPs are associated with significantly higher radiation exposure of patients on the specific procedure. Endoscopists should be aware of the determining factors, including patients with obesity, who underwent mechanical lithotripsy, who had malignant biliary obstruction, and who underwent a procedure using a needle knife, that affect FT during ERCP.

## 1 Introduction

Endoscopic retrograde cholangiopancreatography (ERCP) is an invasive and important procedure that has been used for the diagnosis and treatment of pancreaticobiliary disease. With the recent advancement of noninvasive diagnostic tools, such as magnetic resonance imaging, magnetic resonance cholangiopancreatography, and endoscopic ultrasonography, the need for diagnostic ERCP has been decreasing. On the contrary, the need for therapeutic ERCP has been increasing because of the high number of pancreaticobiliary disease cases.^[1]^

Most ERCPs are performed with fluoroscopic guidance to obtain the images of the biliary tract and pancreas. Fluoroscopy performed during ERCP is associated with an evident risk of radiation exposure for patients, physicians, and assistants,^[2]^ with cancer being the most concerning potential long-term risk caused by radiation,^[3]^ but studies that assess this risk are insufficient. Despite a relatively low risk of radiation-induced injury, physicians should be aware of the cumulative risk from radiation exposure. Reducing radiation exposure is important in reducing its harmful effects on the part of the endoscopists, who perform over several hundred ERCPs per year.

Radiation doses that patients are exposed to while undergoing diagnostic radiological assessments (e.g., simple X-ray, computed tomography scanning) or radiological interventions have been widely monitored clinically. In the clinical field, the indicators of radiation exposure usually include the absorbed dose (AD), which is a measure of radiation concentration, and 2 measures of total radiation (effective dose [ED] and dose-area product [DAP]) and fluoroscopy time (FT). Radiation dose is positively associated with FT, and measuring the FT is simple and important.^[4]^ Reducing the fluoroscopy duration is the most effective and easiest method to minimize radiation exposure during ERCP. However, reducing fluoroscopy duration has been challenging. Measuring the FT has been related to an overall reduction in radiation exposure. Radiation duration is dependent on many factors, including the diagnosis, the type of procedure, the procedure setting (i.e., the diagnostic ERCP or the therapeutic ERCP), the presence of an educational program and policy about radiation exposure, the degree of endoscopic skills and techniques, and the patient factors (e.g., obesity, altered anatomy, and degree of sedation), all of which affect the procedure duration and lead to prolonged use of fluoroscopy.^[4–7]^

Most studies have focused on the effect of radiation exposure during various intervention procedures, such as interventional radiology, coronary angiography, and spinal procedure.^[8–10]^ To the best of our knowledge, there are no clinical studies evaluating radiation exposure in terms of different variables, such as patient factors, procedural factors, and diagnosis during ERCP in Korea. This study aimed to analyze radiation doses the physician is exposed to during ERCP and to identify predictive factors of radiation exposure during the procedure. Additionally, we further evaluated the characteristics of patients and procedural factors that are associated with prolonged FT.

## 2 Methods

### 2.1 Study design

This study was a retrospective analysis of a prospectively collected database of ERCP cases at a tertiary hospital between September 2014 and August 2016. The ERCP database was updated daily using a standardized reporting system. It included patients’ demographic data (e.g., age, gender, height, weight, comorbidity), data on procedure-related characteristics (indication for ERCP, types of procedure, timing of ERCP, and procedure-related adverse events), data on radiation quantities (FT, DAP, AD, and numbers of spot), and data on the procedural endoscopists. All data on the ERCP procedure were recorded, including cannulation time, procedure time, instruments used for the ERCP, and types of procedures performed during ERCP. Incomplete data were retrospectively filled by chart review, and duplicate or incomplete data without documented information were excluded. The study was approved by the institutional review board (Kyung Hee University Hospital, Seoul, South Korea), and informed consent was waived due to the retrospective nature of the study.

### 2.2 Procedure

All ERCPs were performed using a high-definition duodenoscope and gastroscope (TJF-260, JF-260 V, GIF-260J, Olympus, Tokyo, Japan) and 1 fluoroscopy system (Sonialvision Safire II, Shimadzu, Tokyo, Japan). This fluoroscopy system is equipped with an over-couch X-ray tube and collimator, an under-couch image intensifier and image receiver, and a digital image system, allowing automatic brightness/exposure control. The radiation data related to ERCP are automatically recorded by a pre-installed equipment in an ERCP unit. This unit automatically shows fluoroscopy duration. The fluoroscopy system was entirely operated by the attending radiology technician, who had been involved in such procedures for a long time. All ERCPs during the study period were performed by 1 of the 2 expert ERCP physicians (all having performed 1000 ERCPs). The fellows in training did not participate in any of these procedures during the study.

### 2.3 Outcome measurements and statistical analysis

The primary outcome of interest was radiation exposure during ERCP, which was determined by FT. Additionally, the association between variables and patient radiation exposure was determined.

Statistical analyses were performed using the Statistical Package for the Social Sciences (SPSS) software (version 22K, SPSS Korea). *P* values < .05 were considered statistically significant. Data and variables were presented as means (standard deviation), and categorical variables were presented as absolute values and percentages. Continuous variables were tested using analysis of variance with post hoc multiple comparisons. Categorical variables were expressed in the binary form and tested using log-likelihood ratios accordingly. To investigate the impact of radiation exposure on the characteristics of the procedures with respect to the diagnosis, procedure name, and difficulty of procedure, logistic regression analysis was performed after adjusting for age, gender, and body mass index (BMI). Univariate analysis was performed with factors related to the effects on the total FT. Multivariate analysis showed the adjusted *R*^*2*^ using multiple regression analysis.

## 3 Results

### 3.1 Patient characteristics

**Figure 1:**
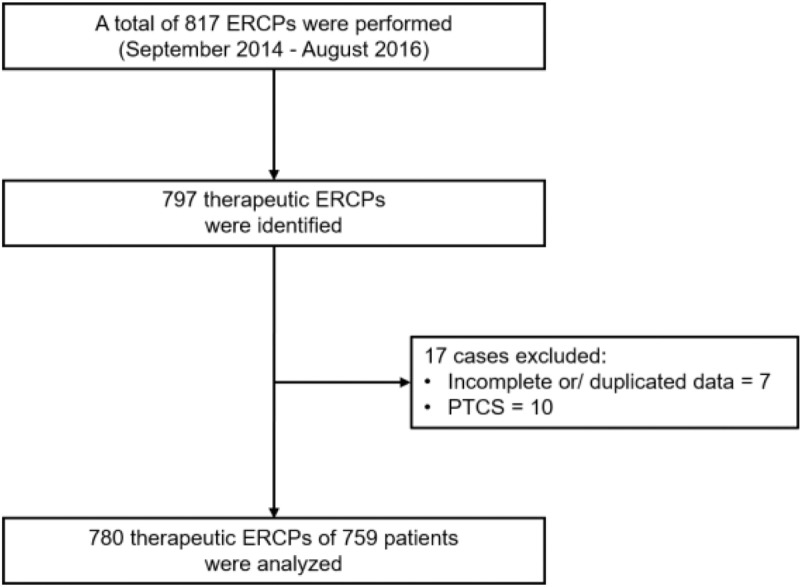
Flowchart of the study. ERCP = endoscopic retrograde cholangiopancreatography, PTCS = percutaneous transhepatic cholangioscopy.

Out of the 817 ERCPs performed, 797 therapeutic ERCPs were considered eligible for our study. Among these, 17 ERCPs were excluded because of incomplete data (n = 7) and percutaneous transhepatic cholangioscopy (n = 10). In total, we analyzed 780 ERCPs in 759 patients (Fig. 1).

The baseline characteristics of patients are summarized in Table 1. The mean (standard deviation) age of the patients was 56.8 ± 17 years, and 58% (n = 440/759) of the patients were men. According to the World Health Organization obesity classification for Asian populations and the previous Korean study,^[11,12]^ patients were subdivided into the following 4 groups: underweight, BMI < 20 kg/m^2^; normal weight, BMI of 18.5–22.9 kg/m^2^; overweight, BMI of 23–27.5 kg/m^2^; and obesity, BMI > 27.5 kg/m^2^. The numbers of subjects in each group were 75 (9.9%), 324 (42.7%), 291 (38.3%), and 69 (9.1%), respectively.

**Table 1:**
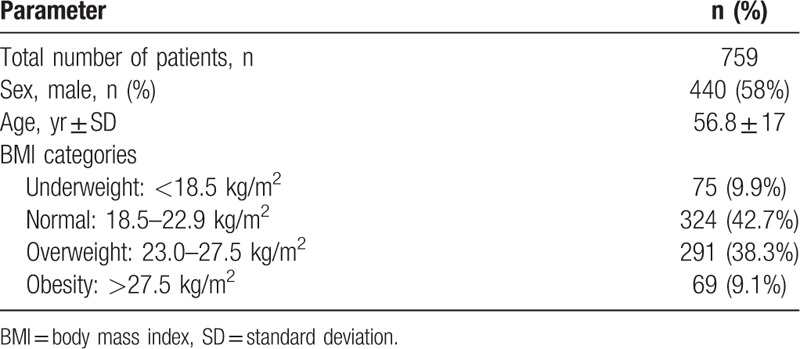
Baseline characteristics of the patients.

### 3.2 Characteristics of the procedures and FT

Table 2 shows the characteristics of the procedures. Major indications for ERCPs included choledocholithiasis (50.6%), malignant biliary obstruction (25.4%), pancreatic disease (10.4%), and benign biliary stricture (10.3%). Out of the 780 ERCPs, endoscopist A and endoscopist B performed 592 ERCPs and 188 ERCPs, respectively.

**Table 2:**
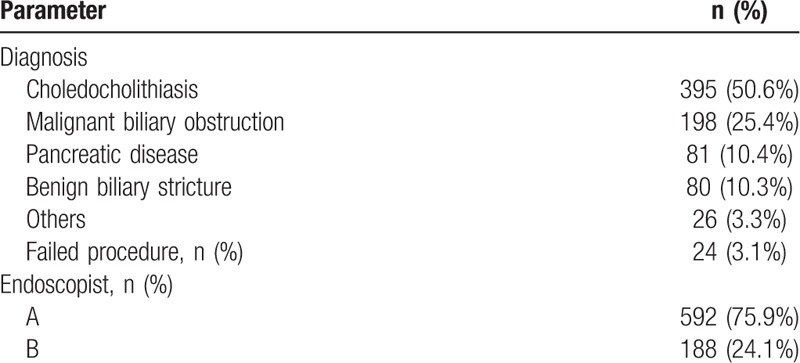
Characteristics and results of endoscopic retrograde cholangiopancreatography.

The mean FT was 5.07 minutes (95% confidence interval [CI], 4.87–5.26). The mean FT based on BMI is shown in Table 3. The mean FT in the obesity group (BMI > 27.5 kg/m^2^) was 5.32 minutes (95% CI, 4.30–6.35), whereas it was 4.97 minutes (95% CI, 3.71–5.98) in the underweight group (BMI < 18.5 kg/m^2^), 4.99 minutes (95% CI, 3.68–6.01) in the normal group (BMI, 18.5–22.9 kg/m^2^), and 5.12 minutes (95% CI, 4.23–6.43) in the overweight group (BMI, 23.0–27.5 kg/m^2^). Additionally, the mean FT of cholelithiasis was 5.76 minutes (95% CI, 4.75–6.80). The mean FT of malignant biliary obstruction was 6.13 minutes (95% CI, 5.91–6.35) (Table 4).

**Table 3:**
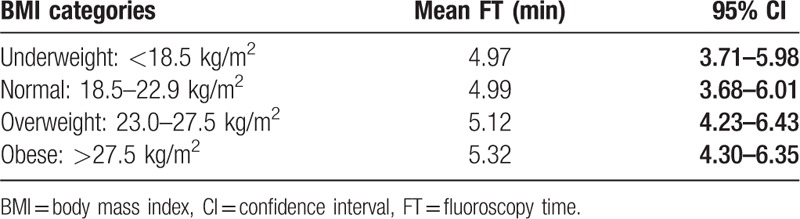
Mean fluoroscopy time and body mass index.

**Table 4:**
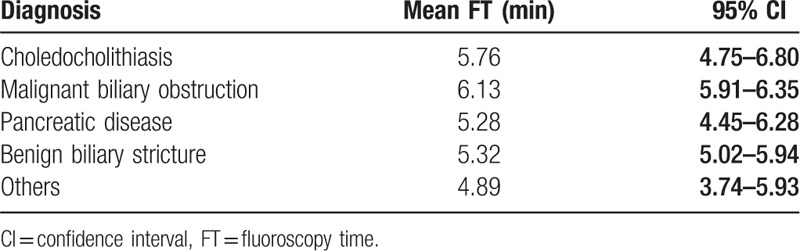
Mean fluoroscopy time and diagnosis.

### 3.3 Variables affecting fluoroscopy duration

After analysis using a univariate model, several variables significantly prolonged the FT (Table 5). These included the following: overweight patient (BMI, 23.0–27.5 kg/m^2^), +3.72 minutes (95% CI, 2.24–5.19; *P* < .0001); obesity patient (BMI > 27.5 kg/m^2^), +4.74 minutes (95% CI, 2.89–6.59; *P* < .0001); malignant biliary obstruction, +4.36 minutes (95% CI, 1.86–6.81; *P* < .0001); pancreatic disease, +3.35 minutes (95% CI, 0.13–6.56; *P* = .035); biliary stent insertion, +3.04 minutes (95% CI, 1.42–4.65; *P* < .0001); intraductal biopsy and/or blush, +4.31 minutes (95% CI, 2.56–6.05; *P* = .042); mechanical lithotripsy, +6.74 minutes (95% CI, 1.95–11.53; *P* = .0001); and needle-knife use, +5.73 minutes (95% CI, 2.15–9.31, *P* = .0018). Significant differences affecting fluoroscopy duration between the 2 endoscopists were not observed. Junior endoscopist (endoscopist A) showed similar fluoroscopy duration to that of the reference endoscopist (endoscopist B; +0.28 minutes; 95% CI, 1.54–2.1; *P* = .233).

**Table 5:**
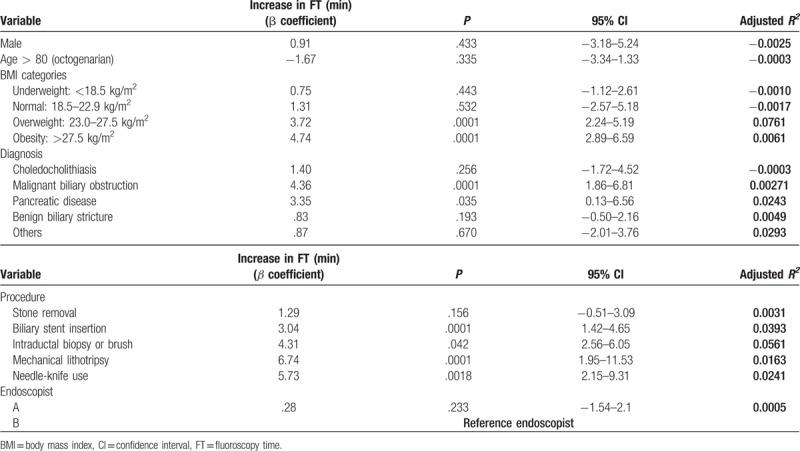
Factors affecting fluoroscopy duration (univariate analysis).

In a multivariate analysis, the variables (Table 6) that were found to significantly increase FT included obesity patient (BMI > 27.5 kg/m^2^; +4.1 minutes; 95% CI, 2.56–5.63), mechanical lithotripsy (+4.85 minutes; 95% CI, 0.45–9.25), needle-knife use (+4.5 minutes; 95% CI, 2.15–6.86), and malignant biliary obstruction (+2.34 minutes; 95% CI, 0.15–4.53).

**Table 6:**
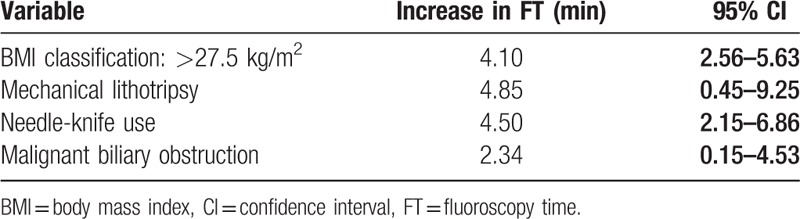
Significant variables in multivariate analysis.

## 4 Discussion

The importance of awareness of radiation exposure during ERCP has been consistently emphasized in the past. The European Society of Digestive Endoscopy developed guidelines for minimizing radiation exposure of patients and physicians during endoscopy.^[13]^ Radiation exposure during ERCP depends on many factors, some of which are impossible to change, including patients, diagnosis, fluoroscopic system, and procedure. Various measures of radiation exposure are used. Because of the convenience of measurement, FT has been widely used to measure radiation exposure during radiological procedures. Previous studies have demonstrated a positive association between radiation exposure and FT.^[4,14,15]^ During ERCP, efforts to minimize FT are strongly recommended. However, factors affecting FT have not been precisely studied. To determine which factors affect radiation exposure time, we evaluated the association between some variables and fluoroscopy duration. In the present study, the factors affecting FT during an ERCP included obesity (higher BMI), malignant biliary obstruction, mechanical lithotripsy, and difficulty in cannulation using a needle knife. These were presumed to be related to the complexity of procedure during ERCP. Moreover, we found that a high BMI significantly affected fluoroscopy duration.

### 4.1 BMI and radiation exposure

Several studies have concluded that higher BMI increases FT, but data on fluoroscopy duration during ERCP are limited.^[16,17]^ In particular, there have been several reports stating that the FT increases as the BMI increases in interventional radiology. Smuck et al^[18]^ showed that the overweight group demonstrated a 30% increase in the mean FT during the spinal procedure for the following reason: when performing the same kind of procedure on obesity patients, the quality of images was not good to confirm the exact position of intervention, and thus, the procedure duration was long. In the present study, we found that FT tended to be longer in the group with higher BMI. Mean FT in the obesity group (BMI > 27.5 kg/m^2^) was 5.32 minutes (95% CI, 4.30–6.35), and according to the multivariate analysis, the obesity group showed longer fluoroscopy duration (+4.1 minutes; 95% CI, 2.56–5.63; *P* < .001).

Obesity patients show reduced sensitivity to sedative agents during ERCPs. Therefore, the procedure time may be longer in obesity patients than patients without obesity due to the former's extra movement during the procedure. Additionally, since fluoroscopy image quality is poor, it is difficult to identify lesions and device locations. It is known that patients with high BMI are exposed to higher radiation doses for a similar quality image, because greater emission from the X-ray tube (generator) needs to pass through a person of higher body mass to reach the image receiver.^[19,20]^ Although recent fluoroscopy systems use an automatic exposure control, which optimizes the dose/image quality ratio to provide the better quality of image, endoscopists should always try to reduce FT when performing ERCP in obesity patients.

### 4.2 Complexity of ERCP and radiation exposure

The effect of various measures of case complexity on FT has been previously discussed. Generally, radiation exposure was greater during therapeutic ERCP than during diagnostic ERCP. In 1 cross-sectional analysis of 269 ERCPs, the mean fluoroscopy duration in therapeutic procedure was significantly higher compared to that in more complex cases, such as in therapeutic procedure (*P* = .002).^[7]^ Additionally, an increase in FT was closely associated with the complexity of the procedure. Therefore, if endoscopists predicted the risk factors for prolonged FT, appropriate precautions could be taken when complex procedures are expected.

Several grading systems for complexity of endoscopic procedures have been suggested. The quality indicators of ERCP by the American Society for Gastrointestinal Endoscopy task force described the level of difficulty of ERCP (Table 6).^[21]^ The later-published Procedure Complexity Score by the American Society for Gastrointestinal Endoscopy working party allowed grading of the procedural complexity.^[22]^ Liao et al^[23]^ suggested the new Stanford Fluoroscopy Complexity Score to account for the number of individual endoscopic interventions to determine the true mandatory radiation exposure for ERCP. These grading systems classified the level of difficulty of ERCP, and it could predict the challenges and outcomes of the procedure.

In our study, we found that complex procedures, such as mechanical lithotripsy (+4.85 minutes; 95% CI, 0.45–9.25), needle-knife use (+4.5 minutes; 95% CI, 2.15–6.86), and malignant biliary obstruction (+2.34 minutes; 95% CI, 0.15–4.53), significantly increased the FT during ERCP. Therefore, effort should be made to reduce FT when performing such a procedure.

### 4.3 Experience of endoscopist

In our study, there was no statistically significant difference in FT between the 2 endoscopists. Each endoscopist had performed at least 1000 ERCPs, and approximately 350 ERCPs are performed each year on average. The more experienced physician did not appear to use less FT. Because gastroenterology (GI) fellows did not participate in the procedure, we could not compare FT according to the degree of training. However, Hoskins and Williams^[24]^ found that radiation exposure decreased as the training duration of trainees increased. In intervention radiology, it has been found that increased levels of training were associated with reduced radiation exposure.^[24]^ In 1 retrospective study, increased experience was directly associated with lower radiation exposure during ERCP training.^[7]^ After performing at least 50 cases of ERCP, the median FT was approximately 2.73 minutes shorter (*P* = .039). As physician or trainees accumulate the numbers of ERCP cases, the radiation time and radiation dose decrease. However, it is unreasonable to compare fluoroscopy duration between physicians because an experienced physician may have performed more difficult and complex procedures compared to a less-experienced physician. We should consider that the association between fluoroscopy duration and the physician's experience may not be consistent. Liao et al^[23]^ reported that ERCPs performed by less-experienced endoscopists were associated with significantly more radiation exposure than those performed by experienced endoscopists. The interesting point in this study is that the FT performed by experienced physician was significantly lower despite the difficulty and complexity of the procedure compared to that performed by less-experienced physicians.

### 4.4 Limitations

The present study has several limitations. First, our study was an observational analysis. Therefore, we could not perform comparative study to investigate the best practices and suggest guidelines to reduce radiation exposure during ERCP. However, retrospective analysis is also meaningful because there may be biases that may arise when the endoscopist is conscious of measuring the FT during a prospective study. Additionally, our study was a retrospective analysis of a single-center ERCP database. We could not explore the wide and exact results of inter-endoscopist and inter-hospital differences in the radiation exposure. Prospective and multicenter comparative trials are also required to standardize the guidelines of radiation exposure during ERCP.

Finally, the present study determined the factors associated with increased radiation exposure using FT as a measure of radiation exposure. As previously mentioned, other exposure indicators, including AD, ED, and DAP, are usually measured in radiological procedures. These measurements can reflect multiple other factors not reflected by FT, including the body size, patient position, and fluoroscopy system. Kachaamy et al^[25]^ suggested that adapting DAP in addition to FT was a good ERCP quality measure to estimate radiation exposure. In addition to FT, we have measured data such as DAP and other measured values. During our study, all ERCP database did not have these parameters such as DAP. Nevertheless, through the analysis of the small ERCP database with the DAP results, we found a strong positive association between FT and DAP. The detailed association between these indicators and variables will be analyzed in the follow-up study. Regarding our findings, several previous studies have shown that FT was associated with radiation exposure.^[4,26–28]^ Therefore, FT is the easiest and most effective measure used by the physician during the procedure. Additionally, in most ERCP centers where DAP measurement is not available, FT can be used as the most important measurement parameter. If a physician is aware of the FT during ERCP, effort to reduce the FT can be continued. Therefore, if the physician remembers the variables previously mentioned and attempts to reduce the FT, radiation exposure during ERCP can be effectively minimized.

In this study, determinant factors including patients with obesity, who underwent mechanical lithotripsy, who had malignant biliary obstruction, and who underwent procedure using a needle knife affected the FT required for ERCP. Based on these predictive factors, ERCP endoscopists could predict the duration of procedures and take appropriate precautions, particularly in complex cases. Prospective comparative trials are required to determine whether FT indicates an increase in the clinically relevant radiation dose. Additionally, studies on other measures of radiation quantities, such as AD, ED, and DAP, are also required.

## Author contributions

**Conceptualization:** Seok Ho Dong.

**Formal analysis:** Chi Hyuk Oh, Jung-Wook Kim.

**Methodology:** Chi Hyuk Oh, Seok Ho Dong.

**Visualization:** Chi Hyuk Oh, Gi-Ae Kim.

**Writing – original draft:** Chi Hyuk Oh, Seok Ho Dong.

**Writing – review & editing:** Jung-Wook Kim, Gi-Ae Kim, Jae Min Lee.
